# *Cntn4*, a risk gene for neuropsychiatric disorders, modulates hippocampal synaptic plasticity and behavior

**DOI:** 10.1038/s41398-021-01223-y

**Published:** 2021-02-04

**Authors:** Asami Oguro-Ando, Rosemary A. Bamford, Wiedjai Sital, Jan J. Sprengers, Amila Zuko, Jolien M. Matser, Hugo Oppelaar, Angela Sarabdjitsingh, Marian Joëls, J. Peter. H. Burbach, Martien J. Kas

**Affiliations:** 1 University of Exeter Medical School, University of Exeter, Exeter, EX2 5DW UK; 2 Department of Translational Neuroscience, Brain Center Rudolf Magnus, UMC Utrecht, Stratenum 4.205, P.O. Box 85060, 3508 AB Utrecht, The Netherlands; 3 Department of Molecular Neurobiology, Donders Institute for Brain, Cognition and Behaviour and Radboud University, Nijmegen, Netherlands; 4 Faculty of Medical Sciences, University of Groningen, Groningen, The Netherlands; 5 Groningen Institute for Evolutionary Life Sciences, University of Groningen, Groningen, The Netherlands

**Keywords:** Neuroscience, Pathogenesis

## Abstract

Neurodevelopmental and neuropsychiatric disorders, such as autism spectrum disorders (ASD), anorexia nervosa (AN), Alzheimer’s disease (AD), and schizophrenia (SZ), are heterogeneous brain disorders with unknown etiology. Genome wide studies have revealed a wide variety of risk genes for these disorders, indicating a biological link between genetic signaling pathways and brain pathology. A unique risk gene is *Contactin 4* (*Cntn4*), an Ig cell adhesion molecule (IgCAM) gene, which has been associated with several neuropsychiatric disorders including ASD, AN, AD, and SZ. Here, we investigated the *Cntn4* gene knockout (KO) mouse model to determine whether memory dysfunction and altered brain plasticity, common neuropsychiatric symptoms, are affected by *Cntn4* genetic disruption. For that purpose, we tested if *Cntn4* genetic disruption affects CA1 synaptic transmission and the ability to induce LTP in hippocampal slices. Stimulation in CA1 striatum radiatum significantly decreased synaptic potentiation in slices of *Cntn4* KO mice. Neuroanatomical analyses showed abnormal dendritic arborization and spines of hippocampal CA1 neurons. Short- and long-term recognition memory, spatial memory, and fear conditioning responses were also assessed. These behavioral studies showed increased contextual fear conditioning in heterozygous and homozygous KO mice, quantified by a gene-dose dependent increase in freezing response. In comparison to wild-type mice, *Cntn4*-deficient animals froze significantly longer and groomed more, indicative of increased stress responsiveness under these test conditions. Our electrophysiological, neuro-anatomical, and behavioral results in *Cntn4* KO mice suggest that Cntn4 has important functions related to fear memory possibly in association with the neuronal morphological and synaptic plasticity changes in hippocampus CA1 neurons.

## Introduction

Neurodevelopmental and neuropsychiatric disorders are a group of heterogeneous brain disorders with unknown etiology. More than 300 million people (4.4%) of the world population suffer from a common mental disorder that involves a suicide attempt, significant work disability, or repeated serious violent behavior^1^. A large number of genome-wide studies have shown that genetic variations, including deletions or duplications, contribute to these disorders by causing imbalances in gene dosage^2^. Interestingly, recent genetic studies have shown several genes that are common in different neuropsychiatric disorders, suggesting that there may be some common phenotype underlying disorders of different psychiatric classifications^3^. To understand the mechanism of how these shared genes are involved in neuropsychiatric disorders may provide insights into how these neurodevelopmental disorders progress.

Genetic variations in neural cell adhesion molecules have been observed across neuropsychiatric disorders. These molecules have important functions for neuronal interactions and supporting neuronal developmental processes including neurite outgrowth and axon guidance^4,5^. Contactin 4 (Cntn4) is one of the proteins belonging to the contactins, a specific subclass of the immunoglobulin CAM superfamily (IgCAM)^6^. These proteins share 40–60% homology at the amino acid sequence level^7^. Contactins are associated with neurodevelopmental processes, for example, *Cntn1* plays an important role in oligodendrocyte maturation and myelination^8^ and *Cntn6* plays a role in dendritic arborization of deep layer cortical neurons and axon branching in the corticospinal tract^9^. Considering the central role of contactins in neurodevelopment this constitutes a potentially interesting group of proteins to investigate in closer depth their functional relation with neuropsychiatric pathology development. This proposal is supported by a previous study identifying certain regions with rare CNVs that were observed in several anorexia nervosa (AN) patients, including CNVs disrupting the *Cntn6/Cntn4* region^10^. *Cntn4* and *Cntn6* have also been reported as candidate risk genes or with associated mutations in Alzheimer’s disease (AD)^11–13^ and schizophrenia (SZ)^14^ with at least one significant SNP found in *Cntn4*^15^.

To understand the neuronal function of *Cntn4*, we require knowledge of expression and localization of Cntn4 in the nervous system. Expression of contactins is observed in the peripheral as well as the central nervous system in rodents^16^, whereas *Cntn4* is expressed in the olfactory bulb, thalamus, hippocampus, and cerebral cortex; sites suggested to play a role in neuropsychiatric phenotypes^17–20^. *Cntn4* has extensive expression in the cortex, namely in layers II-V^17^. Recent single-cell RNA sequencing shows that *Cntn4* is expressed in pyramidal neurons and in *VIP*- and *SST*-expressing interneurons of the cortex and in CA granule cells and interneurons of the hippocampus^21^. CNTN4 is detectable during embryonic development and into adulthood within the axons of olfactory sensory neurons. Functionally, CNTN4 can act as an axon guidance molecule crucial to the proper formation and development of the olfactory and optic systems^22–24^. These expression patterns suggest that *Cntn4* might play a role in the formation of axon connections and support of neural circuits in these regions during the development of the nervous system. *Cntn4* in mice resembles its human orthologue^25^, therefore investigating the anatomical phenotype in knockout mouse models should reveal the role of *Cntn4* in normal and abnormal development.

We present here functional investigations using a *Cntn4* gene knockout mouse model to understand how *Cntn4* loss-of-function impacts brain development and behavior. This study focuses on the hippocampus since Cntn4 is highly expressed in this brain region and because of its involvement in learning and memory performance, a phenotype commonly affected in a wide variety of psychiatric disorders^26–28^. In addition, we begin our understanding of *Cntn4* function in the hippocampus because several IgCAMs have shown to be involved in the development of the dentate gyrus (DG) of the hippocampus. IgCAM deletions have been shown to affect mossy fiber tracts in the hippocampus likely due to fasciculation defects^29–32^, especially since expression is localized specifically in the dentate gyrus granule cells^23^. In addition, previous studies revealed phenotypic differences in the cerebral cortex and hippocampus of *Cntn6*^*-/-*^ mice^33^. *CNTN6* and *CNTN4* are neighboring genes and share over 70% of amino acid identity^34^, all indicating that CNTN proteins may share important functions for neuronal development. In this study, the role of Cntn4 in hippocampal functioning was assessed at the neuro-anatomical, electrophysiological, and behavioral level. Understanding the neurobiological mechanisms underlying brain abnormalities in the hippocampus may eventually contribute to developing clinical treatments with a transdiagnostic application.

## Materials and methods

Full details of materials and methods are provided in the Supplementary Information.

### Animals

*Cntn4*-deficient mice were kindly provided by Dr. Yoshihiro Yoshihara (RIKEN, Japan). These mice were generated using a standard gene-targeting method as previously described^23^. Mice were genotyped at six weeks of age by PCR with extracted DNA and specific primers for *Cntn4* (Table S1). All mice were kept on a normal day/night cycle and had access to food and water *ad libitum* (UMC, Utrecht). All experimental procedures are performed according to the institutional guidelines of the University Medical Center (UMC) Utrecht. All animal procedures were performed according to NIH guidelines and approved by the European Council Directive (86/609/EEC). The rationale of using a male only sample is based on the male predominance of ASD and the potential sex differences.

### Electrophysiology

Healthy 8–12 week old male mice were used for electrophysiology experiments as described previously^35^. One brain slice at a time was moved to a recording chamber with constant perfusion of aCSF (32 °C, flow rate 1.2–1.5 mL/min). Field Excitatory Postsynaptic Potentials (fEPSPs) were recorded in the Schaffer collateral-CA1 pathway as described previously^36,37^. In a separate series of slices a single 100 Hz, 1 s stimulation was applied. Data is pooled for baseline pre-synaptic characteristics 10 Hz and 100 Hz since the data is collected before HFS was applied. Data were acquired, stored, and analyzed using Signal 2.16 (Cambridge 159 Electronic Design, United Kingdom).

### Nissl staining and immunohistochemistry

Nissl staining and immunohistochemistry was carried out as described previously^32^.

### Golgi staining

Golgi staining was performed using a FD Rapid GolgiStain™ kit (FD NeuroTechnologies, Columbia, MD, USA) according to the instructions of the manufacturer.

### Corticosterone assay

Corticosterone levels were determined as previously described^38^.

### Behavior

For behavioral studies, *Cntn4* gene knockout mice were maintained on a C57BL/6 J (Black 6 J) genetic background. Littermate wild type, heterozygous and homozygous *Cntn4* gene knockout animals for behavioral testing were obtained through heterozygous crossings. Measurements were performed during the dark phase of the day, which is the habitual active phase of this nocturnal species, as described previously^39^. A total of 38 male mice, consisting of 12 *Cntn4*^+/+^, 13 *Cntn4*^+/-^, and 13 *Cntn4*^-/-^ mice, were divided into three testing batches of 12 or 13 mice that were randomized for genotype. Behavior tests were carried out as described previously^40^.

### Experimental design and statistical analysis

All experiments were designed to include sample numbers for accurate and appropriate statistical tests, and in accordance to ethical guidelines.

To ensure the experiments had appropriate statistical Power, a Dunn-Sidak correction for the alpha was carried out as follows: 1-[1-0.05]^1/*C*, with *C* the number of comparisons multiplied by the number of parameters. Power analysis is based on the main parameters of error trials during reversal; differences between the means = 3, SD = 2. The number of main parameters (no. of errors, no. of trials to criterion) that need to be corrected for is 2.

All data were analyzed using GraphPad Prism 5 (GraphPad Software, Inc.) For statistical analysis, data is plotted as the mean ± standard error of the mean, unless otherwise stated. Statistical tests were chosen based on data being quantitative and the number of samples. All data was checked prior to statistical tests being carried out that assumptions of the tests were met. Variation within groups of data is estimated by ANOVA output and is checked to be similar between groups of data that are being statistically compared. For all tests, a *P* value <0.05 was considered significant. Heterozygous mice were included where differences between homozygous and wild-type mice were observed. The investigators were blinded to the genotype during experiments and during assessing the out-coming results.

Electrophysiology: paired pulse ratio and baseline synaptic characteristics were analyzed by two-way ANOVA. Synaptic potentiation was analyzed using the unpaired Student’s *t* test and one-way ANOVA. 10 Hz *Cntn4*^+/+^: *n* = 8, *Cntn4*^+/-^: *n* = 6, *Cntn4*^-/-^: *n* = 5 mice. 100 Hz *Cntn4*^+/+^: *n* = 7, *Cntn4*^+/-^: *n* = 9, *Cntn4*^-/-^: *n* = 7 mice.

Nissl staining: statistical analysis between genotypes was performed using unpaired Student’s *t* test and one-way ANOVA. Analysis was performed on at least two sections per brain from *Cntn4*^+/+^, *Cntn4*^+/-^, and *Cntn4*^-/-^ mice (*n* = 4 mice per genotype).

Immunohistochemistry: statistical analysis was performed between genotypes on cell/neuron number and mossy fiber data using unpaired Student’s *t* test and one-way ANOVA. Analysis was performed on at least three sections per brain from *Cntn4*^+/+^ and *Cntn4*^-/-^ mice (*n* = 6 mice per genotype).

Golgi staining: quantitative analysis between genotypes was performed, in each CA1 area, on at least six slices in *Cntn4*^+/+^, *Cntn4*^+/-^, and *Cntn4*^-/-^ mice (*n* = 5 mice per genotype) using the unpaired Student’s *t* test. Neuron data *n* = 19 (*Cntn4*^+/+^), *n* = 14 (*Cntn4*^+/-^), *n* = 14 mice (*Cntn4*^-/-^). Spine data *n* = 10 (*Cntn4*^+/+^), *n* = 14 (*Cntn4*^+/-^), and *n* = 8 mice (*Cntn4*^-/-^). Quantitative analysis was performed between genotypes, in each DG area, on at least six slices in *Cntn4*^+/+^, *Cntn4*^+/-^, and *Cntn4*^-/-^ mice (*n* = 5 mice per genotype) using the unpaired Student’s *t* test and one-way ANOVA. Neuron data *n* = 22 (*Cntn4*^*+*/+^), *n* = 19 (*Cntn4*^+/-^), and *n* = 20 mice (*Cntn4*^-/-^).

Plasma corticosterone levels were analyzed using the unpaired Student’s *t* test and one-way ANOVA (*n* = 31 mice total, *n* = 8 (*Cntn4*^+/+^), *n* = 12 (*Cntn4*^+/-^), and *n* = 11 mice (*Cntn4*^-/-^)).

Behavior: Mice were randomly assigned to behavioral tests. Buried food-seeking (*n* = 38 mice, *n* = 12 (*Cntn4*^+/+^), *n* = 13 (*Cntn4*^+/-^), *n* = 13 mice (*Cntn4*^-/-^)); object location (*n* = 37 mice, *n* = 11 (*Cntn4*^+/+^), *n* = 13 (*Cntn4*^+/-^), *n* = 13 mice (*Cntn4*^-/-^)); object discrimination (*n* = 36 mice, *n* = 11 (*Cntn4*^+/+^), *n* = 13 (*Cntn4*^+/-^), *n* = 12 mice (*Cntn4*^-/-^)), and fear conditioning (*n* = 35 mice, *n* = 11 (*Cntn4*^+/+^), *n* = 12 (*Cntn4*^+/-^), *n* = 12 mice (*Cntn4*^-/-^)). Statistical analysis was performed between genotypes using the unpaired Student’s *t* test and one-way ANOVA.

Gross anatomy: metabolism (*n* = 38 mice, *n* = 12 (*Cntn4*^+/+^), *n* = 13 (*Cntn4*^+/-^), *n* = 13 mice (*Cntn4*^-/-^)); brain size (*n* = 21 mice, *n* = 6 (*Cntn4*^+/+^), *n* = 9 (*Cntn4*^+/-^), *n* = 6 mice (*Cntn4*^-/-^)); brain weight (*n* = 22 mice, *n* = 6 (*Cntn4*^+/+^), *n* = 9 (*Cntn4*^+/-^), *n* = 7 mice (*Cntn4*^-/-^)). Statistical analysis was performed between genotypes using the unpaired Student’s *t* test and one-way ANOVA.

## Results

### Gross anatomy and brain region specific expression

In order to inspect brain development, we first evaluated the gross anatomy in *Cntn4*^-/-^ adult mice. We assessed body weight, total brain weight and total brain size to screen for gross anatomical differences and observed no significant difference between *Cntn4*^-/-^, *Cntn4*^+/-^, and *Cntn4*^+/+^ mice (*p* > 0.05, one-way ANOVA, respectively) (Fig. S1A–C). The expression of Cntn4 is low in the DG based on the single-cell sequencing data of Habib et al.^41^. This was also demonstrated by measuring mRNA expression of *Cntn4* in dissected cortical and hippocampal regions (CA1 and DG) by real-time PCR (RT-PCR) (Fig. S1, D). Expression analysis in wild-type mice revealed that *Cntn4* expression was significantly higher in the CA1 region of the hippocampus, compared to the cortex region. Conversely, *Cntn4* expression is significantly lower in the DG region. Cntn4 protein in cortex and hippocampus extracted from adult male mice was measured by Western blotting, revealing a lack of Cntn4 protein expression in the cortex and hippocampus of the *Cntn4*^-/-^ mice (Fig. S1, E).

### Hippocampal CA1 synaptic potentiation was significantly reduced in *Cntn4*^+/-^ and *Cntn4*^-/-^ mice

To test the role of *Cntn4* in CA1 synaptic transmission and LTP, field excitatory postsynaptic potential recordings were performed in the CA1 region of the hippocampus from *Cntn4*^+/+^, *Cntn4*^+/-^, and *Cntn4*^-/-^ mouse brain slices using a previously described protocol^36^.

Baseline synaptic characteristics, i.e., half maximum slope of the fEPSP and baseline half maximal stimulus intensity were measured and revealed no significant genotype effects (*p* > 0.05, two-way ANOVA) (Table S2).

**Fig. 1:**
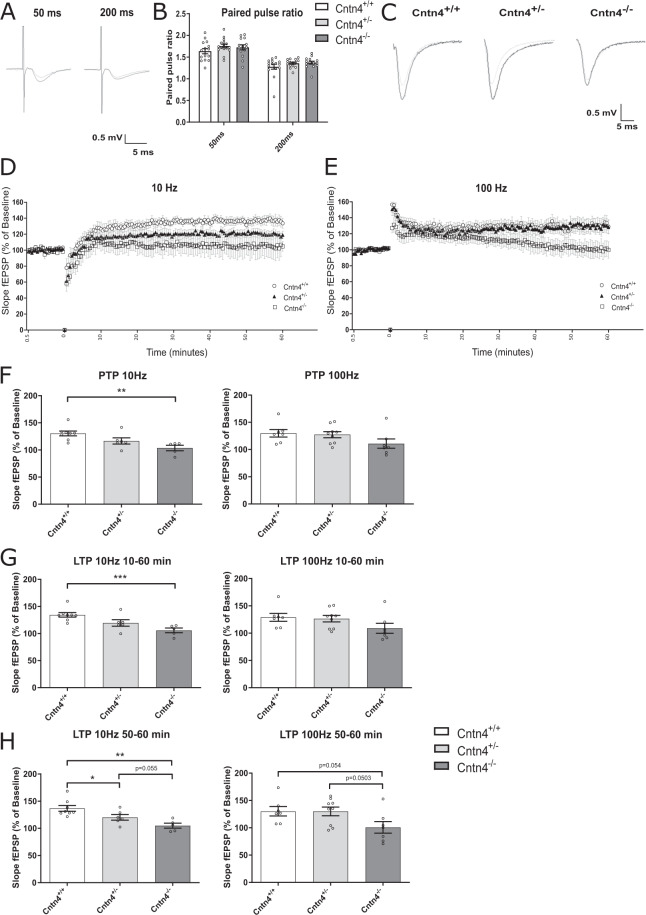
The effect of *Cntn4* deficiency on hippocampal synaptic potentiation. **A** Representative fEPSP traces of the paired pulse ratio (PPR) recorded in the CA1 area of each mouse genotype at 50 ms (left) and 200 ms interval (right). To allow comparison between traces, the response to the first (gray) and second pulse (black) are here superimposed. **B** Paired pulse ratio (expressed as [slope second pulse/slope first pulse]*100%) in the hippocampal region at 50 ms and 200 ms inter-stimulus interval. Data between genotypes was analyzed by two-way ANOVA (*p* = 0.07). **C** Representative individual fEPSP traces taken from each genotype. The gray traces represent the baseline fEPSP, the black trace was taken between 50 and 60 min after tetanic stimulation. **D** Stimulation with 900 pulses at 10 Hz induced synaptic potentiation in the CA1 region of hippocampal slices in all groups. **E** Stimulation with 900 pulses at 100 Hz induced synaptic potentiation in the CA1 region of hippocampal slices in all groups. **F** Average post-tetanic potentiation (PTP) measurements at 10 Hz and 100 Hz, respectively. *p* = 0.003. **G** Average synaptic potentiation over 60 min (i.e., from 10 to 60 min post-tetanus) at 10 Hz and 100 Hz. *P* = 0.0009. **H** Average synaptic potentiation between 50–60 min (i.e., from period between 50 to 60 min post-tetanus) at 10 Hz and 100 Hz. 10 Hz: *Cntn4*^+/+^ vs. *Cntn4*^+/-^ *p* = 0.047; *Cntn4*^+/+^ vs. *Cntn4*^-/-^ *p* = 0.001; *Cntn4*^+/-^ vs. *Cntn4*^-/-^ *p* = 0.055. 100 Hz: *Cntn4*^+/+^ vs. *Cntn4*^-/-^ *p* = 0.0503; *Cntn4*^+/-^ vs. *Cntn4*^-/-^ *p* = 0.054. 10 Hz *Cntn4*^+/+^: *n* = 8, *Cntn4*^+/-^: *n* = 6, *Cntn4*^-/-^: *n* = 5. 100 Hz *Cntn4*^+/+^: *n* = 7, *Cntn4*^+/-^: *n* = 9, *Cntn4*^-/-^: *n* = 7 mice. Data are expressed as means ± S.E.M.

To examine whether *Cntn4* deficiency affected paired pulse facilitation, double pulse responses were recorded at 50 ms or 200 ms intervals. There was no observed genotype effect on paired pulse facilitation, at either interval (*p* > 0.05, two-way ANOVA) (Fig. 1A, B).

In mouse hippocampal slices, stimulation in CA1 stratum radiatum with 900 pulses at 10 Hz or 100 Hz, respectively, yielded synaptic potentiation differences between genotypes, which lasted for at least one hour (Fig. 1D, E). A significant depression in average synaptic potentiation was observed between the *Cntn4*^-/-^ and *Cntn4*^+/+^ mice at 10 Hz but less effect observed at 100 Hz (Fig. 1F–H). For example, the average PTP and LTP fEPSP slope (% of baseline) at 10 Hz was reduced from 130 in *Cntn4*^+/+^ to 100 in *Cntn4*^-/-^ mice (*Cntn4*^+/+^ vs. *Cntn4*^-/-^ *p* = 0.003 and *p* = 0.0009, respectively, unpaired Student’s *t* test, mean baseline vs. mean 60 min post-tetanic period). Recordings were carried out over a 60 min period; and LTP results are compared between two time ranges: the overall 10–60 min period and the final ten minutes (50–60 min period). A stimulation frequency of 10 Hz is commonly used to yield synaptic potentiation, however 100 Hz was also used to allow comparison between early and late LTP. These results indicated that *Cntn4* deficiency had a more pronounced effect on early synaptic potentiation.

### *Cntn4* deficiency leads to an increased hippocampus the CA1-3 surface area

**Fig. 2:**
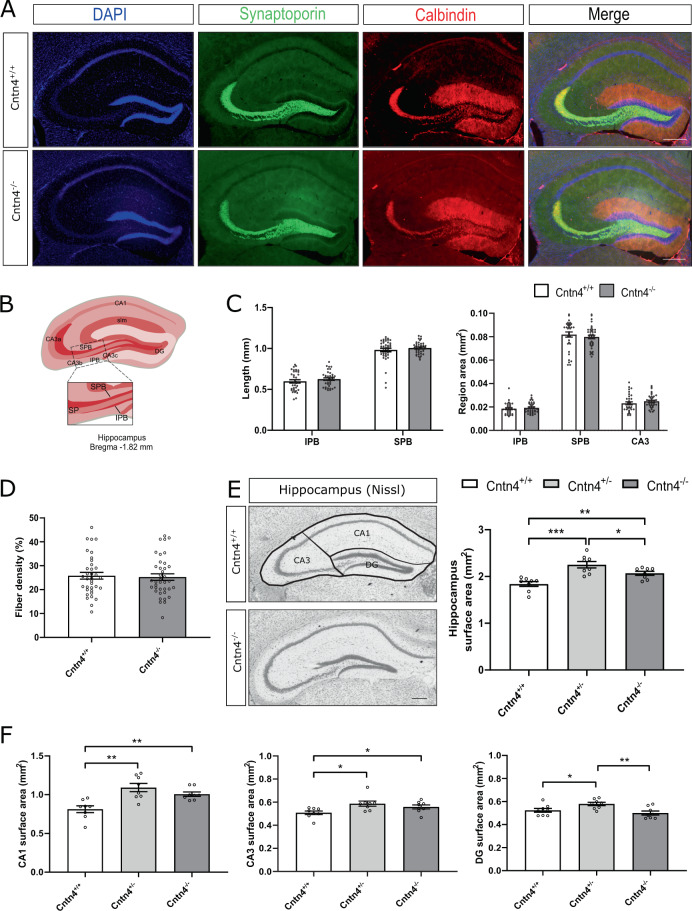
Hippocampal mossy fiber distribution in *Cntn4*-deficient mice. **A** Representative image of synaptoporin (green) and calbindin (red) expression in adult *Cntn4*^+/+^ and *Cntn4*^-/-^ hippocampi. DAPI is in blue. The scale bars represent 250 µm. **B** Schematic representation of the adult mouse hippocampus. The rectangle indicates the area and location used for quantification of mossy fiber crossings in the SP of the CA3. Abbreviations: CA1 cornu ammonis, CA3a-c cornu ammonis 3a-c, DG dentate gyrus, SPB suprapyramidal bundle, IPB infrapyramidal bundle, SP stratum pyramidale, slm stratum lacunosum-moleculare. **C** Quantification of the length (left panel) and area size (right panel) of the IPB, SPB, and CA3 in *Cntn4*^+/+^ and *Cntn4*^-/-^ mice showed no difference between genotypes. Analysis was performed on at least three sections per brain from *Cntn4*^+/+^ and *Cntn4*^-/-^ mice (*n* = 6 mice per genotype) using unpaired Student’s *t* test. Data are presented as mean ± S.E.M. **D** Quantification of percentage of mossy fibers crossing the SP did not reveal a difference between *Cntn4*^+/+^ and *Cntn4*^-/-^ mice. Analysis was performed on at least three sections per brain from *Cntn4*^+/+^ and *Cntn4*^-/-^ mice (*n* = 6 mice per genotype) using unpaired Student’s *t* test. Data are presented as mean ± S.E.M. **E** Nissl-stained sections of *Cntn4*^+/+^, *Cntn4*^+/-^, and *Cntn4*^-/-^ mice demonstrated a significant difference in hippocampal surface areas between genotypes. **F** Tracing of hippocampal subsections revealed significant area differences across all regions. Analysis was performed on at least two sections per brain from *Cntn4*^+/+^, *Cntn4*^+/-^, and *Cntn4*^-/-^ mice (*n* = 4 mice per genotype) using unpaired Student’s *t* test and one-way ANOVA. Data are presented as mean ± S.E.M, *p* = 0.0004, 0.004, 0.04 (total hippocampus); *p* = 0.001, 0.003 (CA1); *p* = 0.01, 0.048 (CA3); *p* = 0.03, 0.004 (DG).

Gross anatomy results interestingly showed CA1 and CA3 area size are significantly increased in the Cntn4-deficient brain, but not in DG (Fig. 2E, F). To investigate whether the Cntn4 knockout phenotype is region specific, we performed immunohistochemistry with two specific markers (synaptoporin and calbindin). These markers were used to highlight the network of hippocampal mossy fibers and their terminals (Fig. 2A). Synaptoporin (also known as Synaptophysin 2) is a component of the synaptic vesicle membrane but is chosen in this context since it is found to be concentrated in the mossy fiber synapses of the hippocampus^42^. Staining shows that synaptoporin was expressed in the mossy fiber system in the hilus of the dentate gyrus (DG) and revealed the suprapyramidal bundle (SPB) and infrapyramidal bundle (IPB)^42^. Simultaneously, staining for the calcium-binding protein calbindin shows DG granular and mossy fiber axons^43^. The mossy fibers cross the stratum pyramidale (SP) of the CA3 region, which emanate from the DG, bifurcate and segregate into the SPB and IPB. Both mossy fiber bundles are located on either side of the SP, which is the layer containing pyramidal neuron somata (Fig. 2B). The density of mossy fiber bundles was measured. Quantification of the length and area size of the hippocampal IPB, SPB, and CA3 was carried out. The lengths and areas of the mossy fibers in IPB, SPB, and CA3 showed no differences between genotypes (*p* > 0.05, one-way ANOVA) (Fig. 2C). The hippocampal mossy fiber distribution was quantified in *Cntn4*-deficient mice. The percentage of mossy fibers crossing the SP did not reveal a difference between *Cntn4*^+/+^ and *Cntn4*^-/-^ mice (*p* > 0.05, one-way ANOVA) (Fig. 2D). Finally, Nissl-stained sections of *Cntn4*^+/+^, *Cntn4*^+/-^, and *Cntn4*^-/-^ mice were analyzed. There is a significant difference in hippocampal surface areas between genotypes (Fig. 2E). *Cntn4*^-/-^ mice showed a bigger hippocampus (on average 2.07 mm^2^) compared to *Cntn4*^+/+^ (on average 1.84 mm^2^) (*Cntn4*^+/+^ vs. *Cntn4*^-/-^ *p* = 0.004, unpaired Student’s *t* test). This was attributed to significant increases in the CA1 and CA3 regions, respectively (Fig. 2F). *Cntn4*^-/-^ mice showed bigger CA1 and CA3 (on average 1.01 mm^2^ and 0.56 mm^2^, respectively) compared to *Cntn4*^+/+^ (on average 0.81 mm^2^ and 0.51 mm^2^, respectively) (*Cntn4*^+/+^ vs. *Cntn4*^-/-^ *p* = 0.003 and 0.048, unpaired Student’s *t* test). No significant difference was observed in cell or neuron number in the hippocampus (*p* > 0.05, one-way ANOVA), aside from an increase in cell number in the DG (Figure S2A-B). These data show a selective enlargement of the hippocampus in animals with *Cntn4* deficiency.

### Increased volume and surface area are found in the CA1 apical dendrites of *Cntn4* deficient mice

**Fig. 3:**
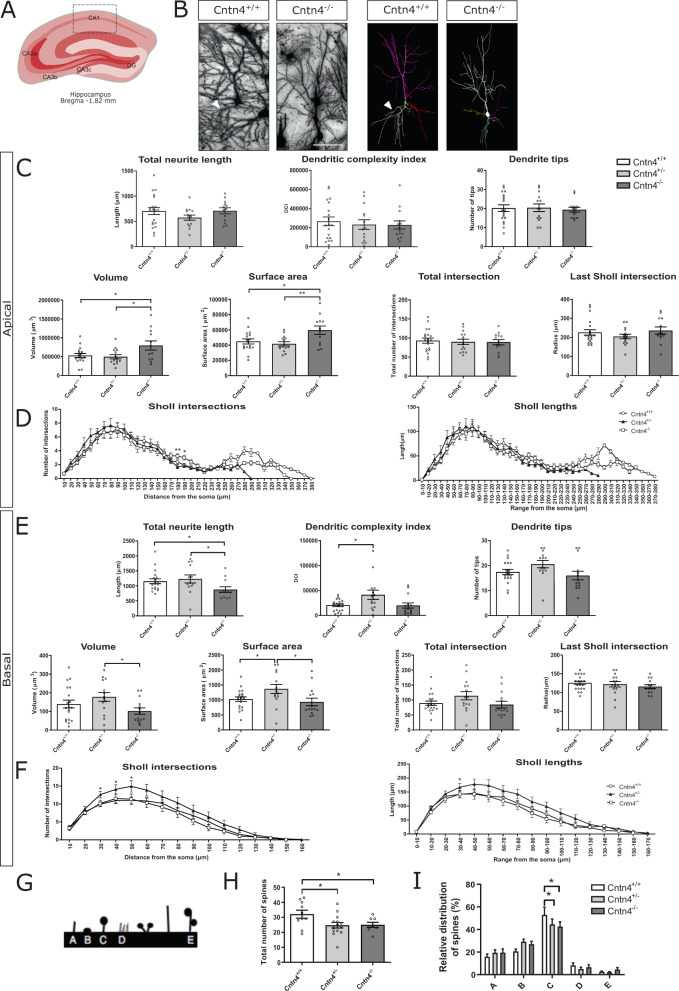
Golgi analysis CA1. Neuron morphology analysis results for *Cntn4*^+/+^, *Cntn4*^+/-^, and *Cntn4*^-/-^ mouse hippocampus CA1. **A** Schematic representation of the hippocampus CA1 with labeled Bregma anterior-posterior. Adapted from Paxinos and Franklin, 2001. **B** Golgi staining in *Cntn4*^+/+^ and *Cntn4*^-/-^ mouse hippocampus CA1 (left), exemplary tracings of pyramidal neurons (right). The scale bar represents 40 µm. The arrowheads show differences in basal neurite length. **C**, **E** Quantitative morphological results for the apical and basal dendrites respectively. *p* = 0.04, 0.03 (apical volume); *p* = 0.02, 0.007 (apical surface area); *p* = 0.04, 0.04 (basal total neurite length); *p* = 0.02 (basal dendritic complexity index); *p* = 0.02 (basal volume); *p* = 0.04, 0.04 (basal surface area). **D**, **F** Sholl plots indicate the distribution of respective apical and basal dendritic intersections and length at increasing distance from the center of the cell body. *p* = 0.004, 0.02 (apical Sholl intersection); *p* = 0.02, 0.05, 0.04 (basal Sholl intersection); *p* = 0.03 (basal Sholl lengths). Quantitative analysis was performed, in each area, on at least six slices in *Cntn4*^+/+^, *Cntn4*^+/-^, and *Cntn4*^-/-^ mice (*n* = 5 mice per genotype). Data are presented as mean ± S. E. M, *n* = 19 (*Cntn4*^+/+^), *n* = 14 (*Cntn4*^+/-^), *n* = 14 mice (*Cntn4*^-/-^). **G** Schematic view of the five different spine morphology categories, A = thin; B = stubby; C = mushroom; D = abnormal (several types); E = double mushroom. Quantitative morphological data on the first 25 μm (50 μm to 75 μm) of Golgi-stained branches of the proximal part of the apical dendrite in pyramidal neurons of the CA1 hippocampus region in *Cntn4*^+/+^, *Cntn4*^+/-^, and *Cntn4*^-/-^ mice indicate a significant difference in **H** total number of spines (including all morphological categories), and **I** relative distribution of mushroom spines. Data are presented as mean ± S. E. M, *n* = 10 (*Cntn4*^+/+^), *n* = 14 (*Cntn4*^+/-^), and *n* = 8 mice (*Cntn4*^-/-^), *p* = 0.04, 0.04 (total spine), *p* = 0.021, 0.029 (mushroom-type spines).

**Fig. 4:**
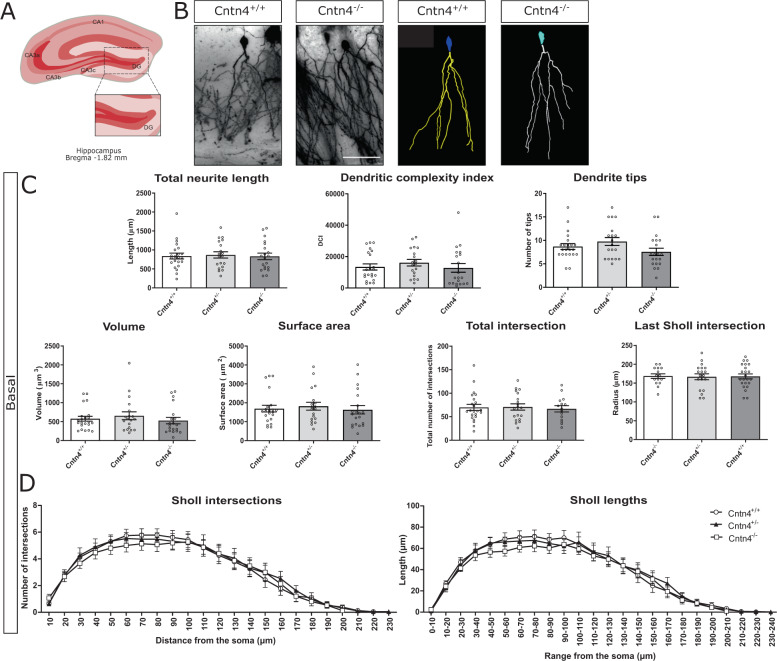
Golgi analysis DG. Neuron morphology analysis results for *Cntn4*^+/+^, *Cntn4*^+/-^, and *Cntn4*^-/-^ mouse hippocampus DG. **A** Schematic representation of the dentate gyrus with labeled Bregma anterior-posterior. Adapted from Paxinos and Franklin, 2001. **B** Golgi staining in *Cntn4*^+/+^ and *Cntn4*^*-*/-^ mice DG (left), exemplary tracings of neurons (right). The scale bar represents 40 µm. **C** Quantitative morphological results for the basal dendrites. **D** Sholl plots indicate the distribution of basal dendritic intersections and length at increasing distance from the center of the cell body. Quantitative analysis was performed, in each area, on at least six slices in *Cntn4*^+/+^, *Cntn4*^+/-^, and *Cntn4*^-/-^ mice (*n* = 5 mice per genotype). Data are presented as mean ± S. E. M, *n* = 22 (*Cntn4*^*+*/+^), *n* = 19 (*Cntn4*^+/-^), *n* = 20 mice (*Cntn4*^-/-^).

Our electrophysiological and morphological analysis revealed that *Cntn4* may be involved in synaptic plasticity, and changes may affect neurons in the hippocampal CA1 region. Subsequently we investigated the morphology of neurons in the CA1 region in detail since *Cntn4* is a key molecule in neural cell connection development. The morphology of pyramidal neurons and granule cells were analyzed in the CA1 and DG regions by quantitative Golgi analyses (Figs. 3 and 4). The apical and basal dendrite morphology of pyramidal neurons in CA1 was quantified from images of Golgi-stained mouse brains (Fig. 3A, B). The apical dendrites of *Cntn4*^-/-^ mice have a significantly larger apical volume and surface area compared to *Cntn4*^+/-^ and *Cntn4*^+/+^ mice (*Cntn4*^-/-^ vs. *Cntn4*^+/-^ *p* = 0.04 and *Cntn4*^-/-^ vs. *Cntn4*^+/+^ *p* = 0.02, unpaired Student’s *t* test) (Fig. 3C). Sholl plots also indicated that the distribution of dendritic intersections and length differed between genotypes. *Cntn4*^-/-^ mice have significantly more Sholl apical dendrite intersections (3.0 ± 0.33) in the range of 180–190 µm from the soma, compared to *Cntn4*^+/-^ (1.82 ± 0.24) and *Cntn4*^+/+^ (1.94 ± 0.26) mice (*Cntn4*^-/-^ vs. *Cntn4*^+/-^ *p* = 0.004 and *Cntn4*^-/-^ vs. *Cntn4*^+/+^ *p* = 0.02, unpaired Student’s *t* test) (Fig. 3D). CA1 basal dendrites were observed to have significantly reduced total neurite length in *Cntn4*^-/-^ mice (878 ± 94 µm) compared to the *Cntn4*^+/-^ (1231 ± 133 µm) and *Cntn4*^+/+^ (1153 ± 84 µm) mice (*Cntn4*^-/-^ vs. *Cntn4*^+/-^ *p* = 0.04 and *Cntn4*^-/-^ vs. *Cntn4*^+/+^ *p* = 0.04, unpaired Student’s *t* test) (Fig. 3E). The dendrites also have significantly smaller volume and surface area in *Cntn4*^-/-^ mice compared to the *Cntn4*^+/-^ mice (Fig. 3E). *Cntn4*^+/-^ mice have significantly more Sholl basal dendrite intersections in the range of 30–50 µm from the soma (12.6–14.9 intersections, respectively) compared to *Cntn4*^+/+^ mice (10–11 intersections, respectively) (*Cntn4*^+/-^ vs. *Cntn4*^+/+^ *p* = 0.02, 0.05, 0.04, respectively, unpaired Student’s *t* test). *Cntn4*^+/-^ mice have significantly longer Sholl basal dendrite lengths in the range 30-40 µm (*Cntn4*^+/-^ vs. *Cntn4*^+/+^ *p* = 0.03, unpaired Student’s *t* test) (Fig. 3F).

Next, spine number and morphology of pyramidal neurons in the CA1 region of the hippocampus were analyzed (Fig. 3G), since spines play a key role in functional neuronal circuits. There was a significant decrease in total number of spines in *Cntn4*^+/-^ (24.7 ± 1.9) and *Cntn4*^-/-^ (25.0 ± 1.7) hippocampi compared to *Cntn4*^+/+^ mice (32.1 ± 2.5) (*Cntn4*^+/-^ vs. *Cntn4*^+/+^ and *Cntn4*^-/-^ vs. *Cntn4*^+/+^ *p* = 0.04, respectively, unpaired Student’s *t* test) (Fig. 3H). Spine morphology analysis was performed to investigate possible changes in spine maturity in *Cntn4* deficient mice. Analysis was focused in the apical dendrites since they project towards the DG, and fEPSP measurements were made here^44,45^. In the first 25 µm (50–75 μm) of the proximal part of the apical dendrite in pyramidal neurons of the CA1 hippocampus region, there was a significant reduction in the number of mushroom spines in *Cntn4*^+/-^ (44.5% ± 4.8%) and *Cntn4*^-/-^ (42.5% ± 4.4%) hippocampi compared to *Cntn4*^+/+^ (53.0% ± 6.8%) mice (*Cntn4*^+/-^ vs. *Cntn4*^+/+^ *p* = 0.021 and *Cntn4*^-/-^ vs. *Cntn4*^+/+^ *p* = 0.029, unpaired Student’s *t* test) (Fig. 3I). In the second 25 μm (75–100 μm) and in the total 50 μm, the total spine number of spines and the spine morphology were similar between genotype mice (*p* > 0.05, one-way ANOVA).

*Cntn4* is expressed less in the DG region compared to CA1. Therefore, to confirm the CA1 phenotype originates from *Cntn4* deficiency, granule cell morphology in the DG was quantified from images of Golgi-stained mice brains (Fig. 4A, B). The granule cell quantitative morphological results, such as the total neurite length, showed no significant differences between genotypes (*p* > 0.05, one-way ANOVA) (Fig. 4C, D).

These data show both neurite and spine dysregulation in the CA1 region, and subsequently raise the possibility that hippocampus-mediated behaviors might have been affected by *Cntn4* deficiency.

### *Cntn4*-deficient mice demonstrate increased fear conditioning behavior

**Fig. 5:**
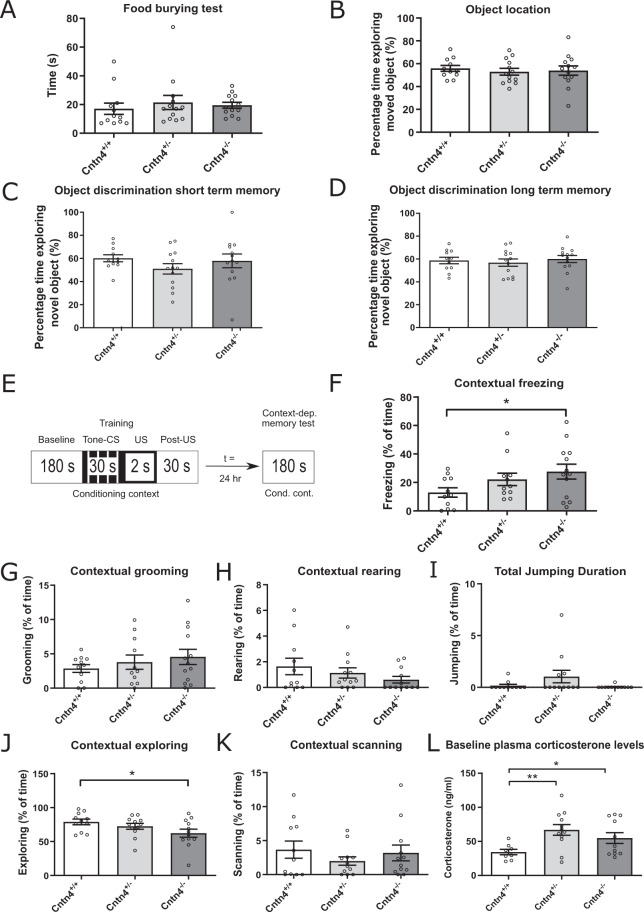
*Cntn4-*deficient mice demonstrate same responses in object discrimination and object location tasks but altered fear conditioning responses. **A** Average time finding the piece of chow (sec) after 24 h of food restriction. The food finding time shows no significant difference between genotypes (*n* = 38 mice). **B** Average exploration time on moved object (%). Calculated as Time_novel_/Time_novel+familiar_. All genotypes spent more time exploring the moved object, however there was no significant difference in exploration time between them (*n* = 37 mice). **C**, **D** Short-term and long-term recognition memory, respectively, in terms of average exploration time on novel object (%). Calculated as Time_novel_/Time_novel+familiar_. All genotypes spent more time exploring the moved object, however there was no significant difference in exploration time between them (*n* = 36 mice). Bars represent the means, error bars indicate the standard error of mean (S.E.M.). **E** Schematic presentation of the fear conditioning test sequence. **F** Percentage of time spent freezing during 180 s of exposure to a conditional context (without simulation such as tone or foot shock). *Cntn4*^-/-^ mice spent significantly more time freezing compared to *Cntn4*^+/+^ mice (*p* = 0.043, *n* = 35 mice). **G**–**I** Percentage of time spent grooming, rearing and jumping during 180 s of exposure to a conditional context (without simulation such as tone or foot shock) revealed no significant genotype effect. There was no significant genotype effect (*n* = 35 mice). **J** Percentage of time spent exploring during 180 s of exposure to a conditional context (without simulation such as tone or foot shock). *Cntn4*^-/-^ mice spent significantly more time exploring compared to *Cntn4*^+/+^ mice (*p* = 0.034, *n* = 35 mice). **K** Percentage of time spent scanning during 180 s of exposure to a conditional context (without simulation such as tone or foot shock) (*n* = 35 mice). **L** Average baseline plasma corticosterone levels (ng/ml) for each genotype. Both the *Cntn4*^+/-^ and *Cntn4*^-/-^ had significantly higher corticosterone levels than the *Cntn4*^+/+^ mice (*p* = 0.035 and *p* = 0.002, respectively; *n* = 31 mice). Bars represent the means, error bars indicate the standard error of mean (S.E.M.).

In the next study, three types of learning and memory tasks were performed to test whether the altered hippocampal size and electrophysiological properties in *Cntn4* deficient mice are related to learning capacity. For that purpose, the following tasks were performed: food latency task, object discrimination task, object location task, and fear conditioning task. First, to evaluate the ability of mice to smell volatile odors, the buried food-seeking task was used. Latencies to find food revealed no significant genotype effect (*p* > 0.05, one-way ANOVA) (Fig. 5A). Second, spatial memory was tested using an object location task. In this task, all genotypes spent more time exploring the moved object. However, there was no significant difference in exploration time between them (Fig. 5B, D) (*p* > 0.05, one-way ANOVA, respectively). Similarly in the object discrimination task no genotype effects were observed (*p* > 0.05, one-way ANOVA). Thus, the altered hippocampal morphology and electrophysiolocal characteristics in *Cntn4*-deficient mice do not seem to be associated with spatial and declarative learning strategies.

Finally, a fear conditioning task was performed to test whether *Cntn4* contributes to associative learning, as described previously^40^. Fear conditioning is based on the association of a neutral (conditioned) stimulus, such as a defined context (multisensory conditioning) or one cue (unisensory conditioning), with an aversive event, for example a foot shock (unconditioned stimulus). Responses, such as freezing, grooming, scanning, jumping, and rearing were monitored during the fear conditioning tests, as this behavior is indicative of anxiety and stress. The full results can be found in Fig. 5 and S3. We found significantly increased contextual fear conditioning in *Cntn4*^-/-^ mice (28% freezing), compared to *Cntn4*^+/+^ mice (13% freezing), quantified by a gene-dose dependent increase in freezing response (*Cntn4*^+/+^ vs. *Cntn4*^-/-^ *p* = 0.043, unpaired Student’s *t* test) (Fig. 5F). However, there was no difference observed between genotypes when exposed to a different context (*p* > 0.05, one-way ANOVA, respectively) (Fig. 5G–K). Finally, in a different context plus a cue (Fig. S3G–K), *Cntn4*^-/-^ mice froze significantly less (40% freezing) than *Cntn4*^+/+^ mice (60% freezing) (*Cntn4*^-/-^ vs. *Cntn4*^+/+^ *p* = 0.01, unpaired Student’s *t* test), but instead spent a significantly higher percentage of time grooming (*Cntn4*^-/-^ vs. *Cntn4*^+/+^ *p* = 0.05, unpaired Student’s *t* test). In addition to the behavioral responses, blood plasma corticosterone concentrations were assessed, in mice which had not undergone any protocols, to investigate whether the contextual fear conditioning responses may be related to changes in basal stress levels. Statistically significant higher levels of baseline blood corticosterone levels were observed in the *Cntn4*^+/-^ (67 ng/ml) and *Cntn4*^-/-^ (55 ng/ml) mice compared to *Cntn4*^+/+^ mice (34 ng/ml) (*Cntn4*^+/-^ vs. *Cntn4*^+/+^ *p* = 0.035, and *Cntn4*^-/-^ vs. *Cntn4*^+/+^ *p* = 0.002, unpaired Student’s *t* test) (Fig. 5L). These data indicate that *Cntn4* deficiency results in selective changes in hippocampus-mediated behaviors, particularly expressed during fear conditioning.

## Discussion

The Cntn4 protein has been characterized as a key cell adhesion molecule for axon guidance and neuronal connection in neuronal development, and previous studies suggest that *CNTN4* is one of the risk genes that is associated with several neuropsychiatric disorders^46^. Here we show that *Cntn4* deficiency contributes to hippocampal CA1 neural circuit morphology, synaptic plasticity, and associative learning. Thus, *CNTN4* genetic mutations may affect hippocampal functionality at the neuro-anatomical, electrophysiological, and behavioral level which is of relevance to the display of maladaptive anxiety responses that are observed in several neuropsychiatric disorders^47–49^.

Our results show that synaptic potentiation was significantly decreased in *Cntn4*^-/-^ mice (Fig. 1D, E). In addition, Golgi analyses revealed abnormal dendritic arborization of hippocampal CA1 pyramidal neurons (Fig. 3). This included increased volume and surface area of CA1 apical dendrites, with the opposite effect in basal dendrites (Fig. 3C). There are increased apical dendrite Sholl intersections at a distance from the cell soma (Fig. 3D). Interestingly, no abnormalities were observed in Golgi analyses of hippocampal DG granule cells (Fig. 4) which is in agreement with the absence of *Cntn4* transcripts in the DG and supporting the argument that the phenotype in CA1 arises from *Cntn4* deficiency^21^. We also examined if *Cntn4* plays a role in the fasciculation of mossy fibers in the hippocampus in adult mouse brain (Fig. 2). This system is sensitive to axon guidance defects or to absence of certain CAMs, as was demonstrated in *Chl1*^-/-^ mice^50,51^. Mossy fibers represent the fasciculated axonal projections of DG granule cells on pyramidal cells in the hippocampus. Their terminals form synapses in the stratum lucidum with the proximal portion of the apical dendrites of CA3 pyramidal cells. *Cntn4* deficiency does not affect the fiber density or morphology in the hippocampus. Since expression of Cntn4 is very low to absent in the DG and highest in the CA1 and CA3 regions based on the single-cell sequencing data [41] and confirmed by our own qPCR data (Figure S1,D), the effect of *Cntn4* deletion on neuronal morphologies would be much smaller in the DG than in the CA1 region. Thus, the DG vs. CA1 comparison on neuronal morphologies is important to show the impact of the *Cntn4*-deficiency, indicating that the morphological changes in the *Cntn4* gene knockout neurons are specific to the lack of Cntn4 expression in this specific hippocampal region (Figs. 3 and 4). This suggests that *Cntn4* regulates the neuronal functions specific in CA1, not in the DG, and supports the notion that *Cntn4* is playing an important role in synaptic plasticity and memory formation in the hippocampus.

Morphological analysis of brains from *Cntn4*-deficient mice CA1 pyramidal neurons revealed that proximal segment spine number and mushroom-type spine number are decreased in *Cntn4*-deficient mice (Fig. 3). Mushroom-type spines have been described as memory spines^52,53^, however they are also an indication of the upper limit synapse size and strength, and have little scope for synaptic strengthening^54,55^. This agrees with our observation that reduced mushroom-type spines are associated with impairments in synaptic potentiation and altered associative learning capacity (Figs. 1 and 5, respectively).

Reduced spine synapse density in the CA1 region of the hippocampus are observed in neuroserpin-deficient mice^56^. Neuroserpin regulates the adhesion protein N-cadherin, which similarly to the Contactin family has been linked to synapse formation^57–59^. Reduced spine density in CA1 hippocampus basal dendrites was also observed in two transgenic amyloid precursor protein (APP) mouse models of Alzheimer’s disease, Tg2576 mice and APP/Lo mice^60^. There are currently limited studies that show a genetic link between *Cntn4* and Alzheimer disease^11–13^. Interestingly, APP has been reported as a Cntn4-interacting protein^61^ and this interaction regulates the promoting target-specific axon arborization in retinal ganglion cells^24^. Although further investigation is necessary, these spine alternations and synaptic potentiation dysfunction may be led by Cntn4/APP protein interaction. In addition, decreased spine density has been associated with altered hippocampal-dependent learning and memory in aged mice^60,62^, whereas strategies that promote spine formation correlate with memory improvement^63^. An association between spine density, hippocampal LTP and memory impairments has also been observed in other Alzheimer disease related animal models^64,65^. Therefore, our results relating a decrease in total number of spines to deficiency in synaptic potentiation and cognitive dysfunction are in line with previous findings.

Cntn4 affects CA1 synaptic transmission and the ability to induce LTP in hippocampal slices. Stimulation in the CA1 stratum radiatum significantly decreased synaptic potentiation in *Cntn4*-deficient mice (Fig. 1D, E). This difference was observed across the induction and maintenance of LTP phases, indicating that there are deficits in both maintenance and induction in the absence of *Cntn4*. There was also a significant difference in the post-tetanic potentiation (PTP), which is strictly a presynaptic phenomenon^66^. It is conspicuous that there are deficits in both LTP and PTP. However, no change in paired pulse ratio (PPR) indicates there is no disruption in the vesicle release of the presynaptic neurons and the type of receptors present at the postsynaptic neuron. These results therefore suggest that *Cntn4* is involved in postsynaptic potentiation, in line with similar reports^67,68^. Reumann et al. observed that LTP differed significantly between neuroserpin-deficient mice and control littermates. The structure and density of dendritic spines correlates with synaptic function, measurable as LTP^69^. Whether the alteration in LTP is cause or consequence of the reduced spine-synaptic number observed in *Cntn4*-deficient mice needs to be further investigated.

Our results show morphological and functional deficits in the hippocampus, leading to the question if this phenotype translates to behavioral deficits, especially related to learning and memory performance. For that purpose, short- and long-term recognition memory, spatial memory and fear conditioning responses were assessed. Buried food-seeking, object location, and discrimination (Fig. 5A–D) did not differ between *Cntn4*^-/-^ and control mice. In the fear conditioning task, genotype differences were observed, indicating that associative learning processes are affected as a function of *Cntn4* deficiency (Fig. 5F–K). The context-dependent test phase of this learning paradigm demonstrates hippocampal impairment, however the cue-dependent test (Figure S3G–K) suggests impairment independent of the hippocampus, possibly the amygdala^70,71^. The cue-dependent test did, however, reveal fear response behavior through significantly increased percentage of time spent grooming. These and earlier results from *Cntn4* gene knockout mice^72^ consistently indicate that the morphological and electrophysiological changes in these mice do have functional consequences at the behavioral level. For example, in addition to the fear conditioning phenotype from the present study, Molenhuis et al. showed increased startle responsiveness and enhanced acquisition in a spatial learning task in *Cntn4*-deficient mice^72^. There is a notable discrepancy between the impaired CA1 LTP and reduction in CA1 spine number, and the longer contextual freezing time. For this translation it is important to realize that Cntn4 is also expressed by other brain regions, like the cortex, which may contribute to the ultimate behavioral response. For example, it is worth noting that the spatial overlap between Cntn4 and Cntn6 expression^32,34^, and evidence of motor impairments caused by Cntn6 deficiency in the cortex^73^, may result in Cntn4 deficiency in the cortex to impact the behavior in the hippocampus. Interestingly, hippocampus morphological and electrophysiological deficits are well documented to be associated with memory and cognition deficits. The present study shows, however, that despite these hippocampal deficits, *Cntn4* does not contribute to general cognitive impairment. Instead, some specific domains related to associative learning seem to be affected. However, considering the increased baseline corticosterone levels in the *Cntn4-*deficient mice, additional experiments are needed to investigate the relationship between *Cntn4* deficiency, stress responsiveness, and hippocampal functioning.

Together, our neuro-anatomical, electrophysiological, and behavioral results in *Cntn4*-deficient mice suggest that *Cntn4* has important functions related to synaptic plasticity and associative learning which occur in association with the neuronal morphological and synaptic plasticity changes in hippocampus CA1 neurons. The results indicate that *Cntn4* plays an important role in pathways that regulate spine morphogenesis, and that dendritic spines could be important substrates of pathogenesis caused by the loss-of-function of *Cntn4*. Our approach will permit future evaluation of how variation in *Cntn4* may act to modulate risk and phenotypic presentation in neuropsychiatric patients with a loss or additional copy of this gene. Brain morphology and histopathology can be an important read-out to identify shared risk factors, and help to unravel the etiology of neuropsychiatric disorders. Further work is required to explain the molecular pathways of *Cntn4* contribution to synaptic plasticity and its behavioral consequences.

## Supplementary information

Supplementary materials

supplementary figure 1

supplementary figure 2

supplementary figure 3
