# Supplementary material for: Cntn4, a risk gene for neuropsychiatric disorders, modulates hippocampal synaptic plasticity and behavior
Source: Transl Psychiatry. 2021 Feb 4;11:106. doi: 10.1038/s41398-021-01223-y (PMC7862349; doi:10.1038/s41398-021-01223-y)
Supplement: Supplementary file 1 — Supplementary materials [file 41398_2021_1223_MOESM1_ESM.docx]

Supplementary Materials and Methods

**Animals**

A targeting vector was designated to mutate the translation start codon (ATG) in the exon 2 of the Cntn4 gene into a stop codon (TAG) and introduce a pgk-neo selection marker. Consequently, these mice were backcrossed with C57BL/6 mice more than nine times. Upon arrival in the University Medical Center Utrecht, the mice were re-derived, followed by heterozygous breeding to generate littermate wild types (*Cntn4*^+/+^), heterozygotes (*Cntn4*^+/-^) and homozygous *Cntn4* gene global knockout mice (*Cntn4*^-/-^) for the use in our experiments.

Genotyping was carried out on 6-week old mice. Briefly, DNA from ear tag was extracted following lysis (0.01M NaOH, 1mM EDTA, Proteinase K), and subjected to PCR using specific primers for *Cntn4* (Table S1) with the resulting products separated and visualized using agarose gel electrophoresis. Genotype was determined by the presence of product molecular weight 1100 bp (*Cntn4^+/+^*), 850 bp (*Cntn4^-/-^*) or the presence of both products (*Cntn4^+/-^*).

For immunohistochemistry, adult male mice were anesthetized with an overdose of sodium pentobarbital (19.4 µl/gr) and were perfused intracardially with 0.9% saline, followed by 4% PFA in phosphate-buffered saline (PBS), pH 7.5. Brains were post fixed in 4% PFA before transferred to 30% sucrose for cryopreservation.

**Quantitative real-time PCR assays of mRNA expression**

The mRNA expression of *Cntn4* in cortex and hippocampus regions (CA1 side and DG side) extracted from adult male mice ^1^ were measured by real-time PCR (RT-PCR) using PrimeScript reverse transcription reagent kit (Takara Bio Group, Japan), as described ^2^. Briefly, total RNA was extracted from fresh brain tissue using Trizol reagent (Invitrogen Corp., CA, USA) and Direct-zol RNA miniprep (Zymo Research, CA, USA) according to manufacturer’s instructions. Total RNA was converted to cDNA using PrimeScript, and RT-PCR was performed using HOT FIREPol EvaGreen (Solis Biodyne, Estonia) on the QuantStudio 6 Flex real-time PCR system (ThermoFisher Scientific, UK). *Gapdh and Hprt1* mRNAs were employed as endogenous controls. The primers employed in these RT-PCR experiments are described in Supplementary Table S1. The relative expression of each test transcript was determined by the comparative Ct approach. Expression levels were calculated relative to the geometric mean of the endogenous controls, but also to the global mean of expression across all transcripts tested, which was empirically determined to vary across test conditions to provide a robust baseline. Expression levels were normalized to the mean of expression seen in cortex. Analysis was performed on three regions (cortex, CA1 side and DG side) from *Cntn4^+/+^* mice (n = 2). Statistical analysis between tissue regions was performed using one-way ANOVA and Tukey’s multiple comparison post-hoc test. Data are expressed as means ± S.E.M.

**Western blotting**

The protein expression of CNTN4 in cortex and hippocampus extracted from adult male mice were measured by Western blotting. Fresh brain tissue was re-suspended in ice-cold lysis buffer (10 mM Tris/HCl pH 7.5, 150 mM NaCl, 0.1% SDS, 1% Triton X-100, 1% Deoxycholate, 0.5 mM EDTA, 1 mM PMSF, Complete protease inhibitor cocktail (Roche, UK), protease inhibitor cocktail (Sigma, UK) and phosphatase inhibitor cocktail 2, 3 (Sigma, UK)), agitated for 30 mins at 4°C, followed by centrifugation at 13,200 rpm for 10 min at 4°C. The supernatant was collected, SDS sample buffer containing 2% β-mercaptoethanol was added and samples were boiled for 5 min at 95°C. Proteins were separated in 7.5% SDS-PAGE gels and transferred onto PVDF membrane (Immobilon-P, Merck Millipore, UK). Membranes were incubated in blocking buffer (TBS, 1% (v/v) Tween 20, 5% milk powder, 5% BSA, 1% FBS) for 1 hour at RT. Membranes were incubated with corresponding primary antibodies in blocking buffer (TBS, 1% (v/v) Tween 20, 2% milk powder) overnight at 4°C. Primary antibodies used: goat anti-Cntn4 (EB11768, Everest, UK) 1:1000 and mouse anti-beta-actin (NB600-501, Novus Biologicals, UK) 1:2500. Secondary antibodies used: donkey anti-goat (IRDye 800CW, LI-COR Biosciences, NE, USA) 1:5000 and goat anti-mouse (DyLight 680, Invitrogen, UK) 1:5000. Blots were imaged using the LI-COR Odyssey CLx (LI-COR Biosciences, NE, USA). Western blotting was performed on two brain regions (cortex and hippocampus) from *Cntn4^+/+^* and *Cntn4^-/-^* mice (n = 2 mice per genotype).

**Electrophysiology**

Brains were placed in ice-cold artificial cerebrospinal fluid (aCSF) containing (in mM): NaCl (120), KCl (3.5), MgSO_4_ (1.3), NaH_2_PO_4_ (1.25), CaCl_2_ (0.5), glucose (10) and NaHCO_3_ (25), continuously gassed with a mixture of 95% O_2_ and 5% CO_2_. Dorsal hippocampal slices (350 µm) were made using a vibratome (LEICA VT 1000S, Germany). Slices were transferred to aCSF containing NaCl (120), KCl (3.5), MgSO_4_ (1.3), NaH_2_PO_4_ (1.25), CaCl_2_ (2.5), glucose (10) and NaHCO_3_ (25) for 20 min at 32°C to recover from the slicing procedure. Subsequently, slices were stored in a continuously carbogenated holding chamber containing aCSF at room temperature for at least 1h before recording commenced.

Field Excitatory Postsynaptic Potentials (fEPSPs) were recorded in the Schaffer collateral-CA1 pathway. In short, a bipolar stimulation electrode (CBBRC75, FHC, USA) was placed on the Schaffer collaterals and a glass recording pipette filled with aCSF (resistance 3–6 MΩ) were positioned in the CA1 stratum radiatum to record fEPSPs.

At the start of the experiment, an input-output curve was established to record the slope of the fEPSP, from which maximal and half-maximal slope as well as the corresponding maximal and half-maximal stimulation intensities were determined (0.067 Hz, stimulus duration 0.15 ms) ^3^. The half-maximal stimulus intensity that was calculated was used throughout the remainder of the recording session. After establishing the input-output curve, we monitored and recorded baseline synaptic transmission using half-maximal stimulation intensity for 10 min (0.033 Hz, duration 0.10 ms). When signals were stable during a baseline period, repetitive tetanic stimulation protocols were applied (10 Hz, 900 pulses).

Post-HFS transmission was recorded for 60 min with the same settings as the baseline recording. At the end of the post-HFS recording, a second input-output was made to determine changes in signal properties. Measurements of PTP were carried out under NMDAR blockade to avoid confounds with LTP.

In a separate series of slices, paired pulse stimulation (at stimulus intervals of 50 ms or 200 ms) was applied at half maximal stimulus intensity and fEPSPs were recorded.

Baseline and high-frequency induced synaptic characteristics, and paired pulse facilitation were analyzed by two-way ANOVA. In case of main or interaction effects, planned post-hoc pairwise group comparisons were carried out, and corrected with Bonferroni for multiple testing. Paired pulse facilitation was expressed as [(slope of the second fEPSP/slope of the first fEPSP)*100%] and synaptic potentiation as fEPSP slope % of baseline.

**Nissl staining**

Brains were sectioned with a cryostat (Leica Microsystems, Wetzlar, Germany) coronally at 40 µm from rostral to caudal. Sections were mounted onto Superfrost slides (VWR, 631-0108). The slices were rehydrated in graded levels of decreased concentrations of alcohol and then stained in 0.5% Cresyl-Violet (Sigma Aldrich, St Louis, MO, USA) for 5 minutes. Finally, the slices were dehydrated in graded levels of increased concentrations of alcohol, cleaned in xylene, and then cover slipped using Entellan® (Merck, Damstadt, Germany).

Slices were imaged using light microscopy (Zeiss Axio Scope.A2, Germany). Imaging was carried out using the following stereotaxic coordinates ^4^: Bregma anterior-posterior -1.82 mm (hippocampus). Hippocampus area was measured on at least two slices in Cntn4^-/-^, Cntn4^+/-^and Cntn4^+/+^ mice (n = 4 mice per genotype) using ImageJ software ^5^.

**Immunohistochemistry**

Brains were sectioned as described previously and free-floating slices were stored in 0.02% sodium azide until immunohistochemistry was performed. The sections were washed with PBS and incubated in blocking buffer (1% BSA, 0.2% fish skin gelatin (Sigma G7765), 0.1% Triton X-100 in PBS) for 45 min. Sections were washed and incubated in permeabilization buffer (0.3% Triton X-100 in PBS) for 10 min before incubation with primary antibody in blocking buffer at 4°C for 2 hr. The sections were washed in PBS and pre-incubated with blocking buffer before incubating with secondary antibody at RT for 2 hr. The sections were embedded with Polyvinyl alcohol mounting medium with DABCO® anti-fading (Fluka, 10981) onto glass slides after additional PBS wash steps.

Primary antibodies were used as follows: Rabbit anti-Synaptoporin (1:1000, Synaptic Systems), mouse anti-Calbindin (1:3000, Swant), mouse anti-NeuN (1:200, Millipore) and DAPI. Appropriate secondary antibodies were used from the Molecular Probes Alexa Series (1:250, Invitrogen).

Images were captured by confocal laser scanning microscopy (Zeiss Axio Scope.A1, Germany) and image analysis carried out in ImageJ. Cells positive for NeuN and DAPI were counted in images taken of the hippocampus. Hippocampus area was measured on sections between -1.5 and -2.5 mm Bregma anterior-posterior. Anti-calbindin was used to visualize mossy fibers and anti-synaptoporin was used to visualize mossy fiber synapses in the hippocampus. The lengths and area sizes of the supra- and infrapyramidal bundles (SPB and IPB) were assessed by tracing the bundles from the endpoints of the dentate gyrus (DG) blades to the last visible bundle staining at the CA3 side. The analytical region of the stratum pyramidale (SP) was assessed by placing a rectangle (Figure 2B) from the endpoints of the DG blades the to the last visible staining of the IPB at the CA3 side. The SP region in this rectangle was traced and both the area size and fiber density were measured. An average measurement from at least five slices was performed in *Cntn4*^-/-^and *Cntn4*^+/+^ mice (n = 6 mice per genotype) using ImageJ software. At least 28 randomly selected microscope fields from the hippocampus were used for all measurements. Statistical analysis was carried out using unpaired Student’s *t* test between genotypes and one-way analysis of variance (ANOVA) used for comparison between two groups.

**Golgi staining**

After treatment with Golgi solutions A, B and C, brains were sectioned with a vibratome (Leica Microsystems, Wetzlar, Germany) coronally at 150 µm from rostral to caudal. The slices were attached to gelatin coated slides and stained with Golgi solutions D + E. Finally, the slices were dehydrated in graded levels of increased concentrations of alcohol, cleaned in xylene, and then cover slipped using Entellan® (Merck, Damstadt, Germany).

Slices were imaged using light microscopy (Zeiss Axio Scope.A2, Germany). Areas assessed were found using the following stereotaxic coordinates ^4^: Bregma anterior-posterior -1.82 mm (hippocampus). Image analysis was carried out with Golgi Microscope (Zeiss AxioImager M2, Germany) and Neurolucida software (MicroBrightField, Williston, VT, USA). Further slices were imaged using light microscopy (Leica TCS SP8, Leica Microsystems, Mannheim, Germany). Areas assessed were from distinct brain regions: Bregma anterior-posterior -1.82 mm (dentate gyrus) and -1.82 mm (CA1) according to Paxinos and Franklin (2001). Image analysis was carried out across serial sections with ImageJ and Neurolucida software (MBF Bioscience, VT, USA).

To study the structural differences in spine number and spine morphology caused by *Cntn4*^-/-^ mice, a total of 16 samples of different mice were included for final analysis: 5 *Cntn4*^+/+^, 7 *Cntn4*^+/-^, 4 *Cntn4*^-/-^ mice (n ≥ 4 animals per group). A total of 32 pyramidal neurons were included for final analysis (10 *Cntn4*^+/+^, 14 *Cntn4*^+/-^, 8 *Cntn4*^-/-^ neurons). Thin, mushroom, stubby, abnormal and double mushroom spines were counted in the first 25 μm (50 μm to 75 μm) and second 25 μm (the 75 μm to 100 μm) of a branch of the proximal part of the apical dendrite (the first 1/3 part of the apical dendrite) in pyramidal neurons of the CA1 region. The total number of spines includes all morphological categories. Neurolucida and Neuroexplorer were used for the tracing of spines and analysis (spine number and spine morphology). The different spine morphology categories were counted. Primary branches at the proximal part (the first 1/3 of the apical dendrite) were included for spine analysis and images acquired including circles with a diameter of 200 µm, 150 µm and 100 µm from the branching place. Branches that were not long enough, or with branching places at the branch itself (between 100 and 200 µm) were not included.

**Corticosterone assay**

Blood samples were determined from *Cntn4*^+/+^, *Cntn4*^+/-^ and *Cntn4*^-/-^ mice which had not undergone any protocols. Trunk blood was collected early in the morning, as described previously ^6^, in heparin-coated tubes and centrifuged (10,000 rpm, 10 min at room temperature). Plasma was stored at -20°C until corticosterone was determined using a commercially available radioactive immunoassay according to the manufacturer’s instructions (RIA; MP Biomedicals Inc., Santa Ana, CA, USA). Corticosterone levels in blood may be used as a marker of stress in rodents ^7^, and were determined as described before ^8^.

**Behavior**

After weaning the mice at three to five weeks after birth, the animals were housed in groups of up to five same sex littermates. Between ten to fourteen weeks after birth (starting one week prior to the experimental battery), the animals were kept in a controlled 12 hour light/dark cycle, with food and water available *ad libitum*. Each mouse of each batch participated in all four tasks of the experimental battery in the order described below. The battery lasted in total for four weeks, after which blood samples were taken, and brains were collected for brain weight, brain size and further structural analysis.

**Object discrimination task**

The object discrimination task consisted of three testing phases: T0, T1 and T24. Testing took place in a macrolon cage type III, without sawdust. During the habituation trial (T0 testing phase), two similar objects (glass, metal or plastic) were placed in the test cage for a duration of five minutes. These objects would be recognised as the familiar object in the following testing phases. After a short-term interval of one hour, each mouse was re-exposed to one of the familiar objects and one novel object for five minutes (short term memory, T1). On the second day, 24 hours after the testing phase, the animals were again exposed to the familiar object and another novel object for five minutes (long term memory, T24). The times spent exploring the objects was manually scored using The Observer XT (Noldus Information Technology, Wageningen, The Netherlands). Active exploration (approaching, sniffing, touching) of the objects, but not passive contact (sitting next to the object) was scored as object exploration. The percentage time exploring the novel object relative to the total object exploration time was used as behavioral endpoint for this task.

**Object location task**

The object location task consisted of two testing phases: T0 and T24, as described previously ^9^. Testing took place in empty transparent plastic cages (26x26x35 cm) with two transparent walls, and two white walls with a black circle on one wall. A black and white object was placed outside the cage (10 cm) distance to provide spatial cues. Two small closed plastic bottles filled with water were used as objects. In the habituation trial (T0 testing phase), the mice were introduced for ten minutes to two identical objects placed in adjacent corners. On the second day, 24 hours after the habituation trial, each mouse was re-exposed for ten minutes to the same objects. However, in this T24 phase, one object was moved to an opposite corner. During all experimental phases, the behaviors were recorded and subsequently manually scored using The Observer XT (Noldus Information Technology, Wageningen, The Netherlands). Calculation of the behavioral endpoint for this task was similar as described for the Object Discrimination task.

**Buried food-seeking task**

For this task, mice were exposed to a single event of food restriction to stimulate the animals start searching for the hidden food. Prior to the testing (18 to 24 hours) all the chow was removed from the animals home cages, and the body weight of the animals was measured. The test food was a piece of chow in order for the animals to recognize the smell of food. Testing took place in macrolon cages type III, with extra sawdust. On the testing day, the animals were weighed again prior to the task (if the mice lost more than 20% of their body weight due to the food restriction they were removed from the task). On average, the mice lost 11.9% of their body weight after the episode of food restriction. There was no significant difference in weight loss between genotypes. After the habituation phase of five minutes, the mice were transferred to a similar cage containing a piece of chow that was hidden approximately 1 cm beneath the surface in a random corner. The time finding the piece of chow was measured with a stopwatch. Latency (time in seconds) to find the buried piece of chow, and the percentage of weight loss, was measured and analyzed for the ability to smell volatile odors.

**Fear conditioning task**

The fear conditioning task was divided into two testing phases: T0 and T24. Testing took place in 30*30*40 cm cages with stainless steel walls, with Phenotyper top units to apply the sound cue and record the behavior. The T0 testing phase on the first day is also referred as training day 1. In this phase, the association between a conditioned stimulus (buzzer) and an unconditioned stimulus (foot shock) was learned. At this training phase, the mouse explored the first testing cage (old context) for 180 seconds, followed by a 30 second buzzer (~2300 Hz; level ~70 dB). Directly after this buzzer, a two second foot shock was given (0.7 mA, constant current), provided through the stainless steel floor grid (Ø1 mm, distance 5 mm). The trial ends with another 30 second exploration period. On testing day 2 (after 24 hours), the animal was re-exposed to the old context for 180 seconds of exploration without any stimulation such as tone or foot shock, to gain contextual memory. Hereafter, the mouse was a placed in a second testing case (new context) to test the tone dependent memory. This new context is a similar cage with a plain floor instead of the shock grid, a black and white wall, and honey flavored smell. After an exploration period of 180 seconds had taken place, there followed the cued trial with 180 seconds of the buzzer (~2300 Hz; level ~70 dB). The activities of the animals were recorded and subsequently manually scored using the Observer software. Behaviors scored consisted of percentage of time spent freezing (active suppression of ongoing behavior defined as the lack of any movement besides respiration) and scanning (not walking, but small head movements). Freezing is defined as a real fear response, while scanning is not. The time spent freezing and showing ‘other’ behavior is compared between the experimental groups in the contextual and cued fear conditioning tests.

**References**

1 Zhang M *et al.* Propagation of Epileptiform Activity Can Be Independent of Synaptic Transmission, Gap Junctions, or Diffusion and Is Consistent with Electrical Field Transmission. *J Neurosci*. **34**, 1409–1419 (2014).

2 Oguro-Ando A *et al.* Increased CYFIP1 dosage alters cellular and dendritic morphology and dysregulates mTOR. *Mol Psychiatry*. **20**, 1069–78 (2015).

3 Wiegert O, Joels M, Krugers H. Timing is essential for rapid effects of corticosterone on synaptic potentiation in the mouse hippocampus. *Learn Mem*. **13**, 110–113 (2006).

4 Paxinos G, Franklin KBJ. *The Mouse Brain in Stereotaxic Coordinates (Deluxe Edition)*. Elsevier Inc.: San Diego, United States, 2001.

5 Schneider CA, Rasband WS, Eliceiri KW. NIH Image to ImageJ: 25 years of image analysis. *Nat Methods*. **9**, 671–5 (2012).

6 Haché RJG, Tse R, Reich T, Savory JGA, Lefebvre YA. Nucleocytoplasmic Trafficking of Steroid-free Glucocorticoid Receptor. *J Biol Chem*. **274**, 1432–1439 (1999).

7 Gong S *et al.* Dynamics and correlation of serum cortisol and corticosterone under different physiological or stressful conditions in mice. *PLoS One*. **10**, e0117503 (2015).

8 Sarabdjitsingh RA, Meijer OC, de Kloet ER. Specificity of glucocorticoid receptor primary antibodies for analysis of receptor localization patterns in cultured cells and rat hippocampus. *Brain Res*. **1331**, 1–11 (2010).

9 Molenhuis RT, de Visser L, Bruining H, Kas MJ. Enhancing the value of psychiatric mouse models; differential expression of developmental behavioral and cognitive profiles in four inbred strains of mice. *Eur Neuropsychopharmacol*. **24**, 945–954 (2014).

Table 1: Primer sequences

|  | Gene | Forward Primer (5’ to 3’) | Reverse Primer (5’ to 3’) |
| --- | --- | --- | --- |
| *i)* | *Cntn4* | TGGTAGATGGATCGATGGCAAACATG  AGCCCCAGTTTTTGCCTAAGCAT | TTCATCACTCCTGAATCACACATGTCA |
| *ii)* | *Cntn4* primer set 1 | CTTTGAAAGAGTTGGAGGGCAGGATT | AGGTCCTGGTCTCCAAGAAAGCTG |
|  | *Cntn4* primer set 2 | Sigma KiCqStart® SYBR®Green Primers. KSPQ12012G, FM1_Cntn4, Species (mouse), Primer Pair ID 1: M_Cntn4_1 | Sigma KiCqStart® SYBR®Green Primers. KSPQ12012G, FM1_Cntn4, Species (mouse), Primer Pair ID 1: M_Cntn4_1 |
|  | *Gapdh* | GCACAGTCAAGGCCGAGAAT | GCCTTCTCCATGGTGGTGAA |
|  | *Hprt1* | GCTCGAGATGTCATGAAGGAGA | TCAGTGCTTTAATGTAATCCAGC |

Primer sequences used for *i)* determining *Cntn4* genotype in mice; and *ii)* quantifying *Cntn4* mRNA expression in mice using the PrimeScript reverse transcription kit (Takara Bio Group).

Table S2: Baseline synaptic characteristics. Data between genotypes was analyzed by two-way ANOVA (Half Max Slope p = 0.6291; Half Max SI p = 0.1840).

| **Baseline pre-synaptic characteristics** | | | |
| --- | --- | --- | --- |
| **Group** | **N** | **Half Max Slope (mV/ms)** | **Half Max SI (mV)** |
| ***Cntn4*^+/+^** | 15 | -525.5 ± 22.5 | 1.86 ± 0.02 |
| ***Cntn4*^+/-^** | 15 | -542 ± 24 | 1.82 ± 0.01 |
| ***Cntn4*^-/-^** | 13 | -498 ± 28 | 1.82 ± 0.01 |
| **Baseline post-synaptic characteristics 10 Hz** | | | |
| **Group** | **N** | **Half Max Slope (mV/ms)** | **Half Max SI (mV)** |
| ***Cntn4*^+/+^** | 8 | -533.5 ± 52 | 1.82 ± 0.01 |
| ***Cntn4*^+/-^** | 6 | -567.5 ± 21 | 1.81 ± 0.03 |
| ***Cntn4*^-/-^** | 4 | -610.5 ± 87 | 1.81 ± 0.05 |
| **Baseline post-synaptic characteristics 100 Hz** | | | |
| **Group** | **N** | **Half Max Slope (mV/ms)** | **Half Max SI (mV)** |
| ***Cntn4*^+/+^** | 7 | -572 ± 34.5 | 1.86 ± 0.04 |
| ***Cntn4*^+/-^** | 7 | -579.5 ± 36.5 | 1.81 ± 0.03 |
| ***Cntn4*^-/-^** | 7 | -607.5 ± 38.5 | 1.85 ± 0.02 |

**Figure S1: General results for mice with *Cntn4* deficiency.**

A) Average weight loss (%) after 24 hours of food restriction. The percentage of weight loss shows no significant difference in metabolic rate between the genotypes (p = 0.6421, n = 38 mice). B) & C) Average brain size and weight of adult mice. In both cases, there is no significant difference between genotypes (p>0.05, n = 21 mice (brain size) and n = 22 mice (brain weight). D) Fold change of *Cntn4* expression levels in hippocampus regions (CA1 side and DG side) compared to cortex, generated by qRT-PCR. *Cntn4* expression are significantly increased in the CA1 side and reduced in the DG side. Analysis was performed on three regions per brain from *Cntn4*^+/+^ mice (n = 2) using one-way ANOVA and Tukey’s multiple comparison post-hoc test. E) Protein extracted from adult *Cntn4*^+/+^ and *Cntn4*^-/-^ cortex and hippocampus were analyzed by Western blot. Blots stained with anti-Cntn4 antibody revealed expression only in *Cntn4*^+/+^ mice. Western blotting was performed on two regions per brain from *Cntn4^+/+^* and *Cntn4^-/-^* mice (n = 2 mice per genotype). Molecular weights are as follows: Cntn4 = 150 kDa; beta-actin = 47 kDa. Data are presented as mean ± S.E.M**.**

**Figure S2: Cell and neuron number unchanged in hippocampus.**

Cell and neuron counting using A) DAPI and B) NeuN staining, respectively, in adult *Cntn4*^+/+^ and *Cntn4*^-/-^ hippocampi. Abbreviations: CA1, cornu ammonis; CA3, cornu ammonis 3; DG, dentate gyrus. Analysis was performed on at least three sections per brain from *Cntn4*^+/+^ and *Cntn4*^-/-^ mice (n = 3 mice per genotype) using unpaired Student’s t test and one-way ANOVA. Data are presented as mean ± S.E.M., p = 0.001**.**

**Figure S3: *Cntn4*-deficient mice demonstrate different responses to the fear condition test.**

A) Schematic presentation of the fear conditioning test sequence. B) Percentage of time spent freezing during 180 seconds of exposure to a new context (without simulation such as tone or foot shock) (p = 0.01, n = 35 mice). There was no significant difference between genotypes. C) Percentage of time spent grooming during 180 seconds of exposure to a new context (without simulation such as tone or foot shock). There was no significant difference between genotypes. D) Percentage of time spent rearing during 180 seconds of exposure to a new context (without simulation such as tone or foot shock). *Cntn4*^-/-^ and *Cntn4*^+/-^ mice spent significantly less time rearing compared to *Cntn4*^+/+^ mice (p = 0.002 and p = 0.02, respectively, n = 35 mice). E) Percentage of time spent exploring during 180 seconds of exposure to a new context (without simulation such as tone or foot shock). There was no significant difference between genotypes. F) Percentage of time spent scanning during 180 seconds of exposure to a new context (without simulation such as tone or foot shock). There was no significant difference between genotypes. G) Percentage of time spent freezing during 180 seconds of exposure to a new context with buzzer. *Cntn4*^-/-^ mice spent significantly less time freezing compared to *Cntn4*^+/+^ mice (p = 0.01, n = 35 mice). H) Percentage of time spent grooming during 180 seconds of exposure to a new context with buzzer. *Cntn4*^-/-^ and *Cntn4*^+/-^ mice spent significantly more time grooming compared to *Cntn4*^+/+^ mice (p = 0.05 and p = 0.04, respectively, n = 35 mice). I) Percentage of time spent rearing during 180 seconds of exposure to a new context with buzzer. There was no significant difference between genotypes. J) Percentage of time spent exploring during 180 seconds of exposure to a new context with buzzer. There was no significant difference between genotypes. K) Percentage of time spent scanning during 180 seconds of exposure to a new context with buzzer. *Cntn4*^-/-^ and *Cntn4*^+/-^ mice spent significantly more time scanning compared to *Cntn4*^+/+^ mice (p = 0.03 and p = 0.05, respectively, n = 35 mice). Bars represent the means, error bars indicate the standard error of mean (S.E.M.).
