# Supplementary figures and images for: Cntn4, a risk gene for neuropsychiatric disorders, modulates hippocampal synaptic plasticity and behavior
Source: Transl Psychiatry. 2021 Feb 4;11:106. doi: 10.1038/s41398-021-01223-y (PMC7862349; doi:10.1038/s41398-021-01223-y)

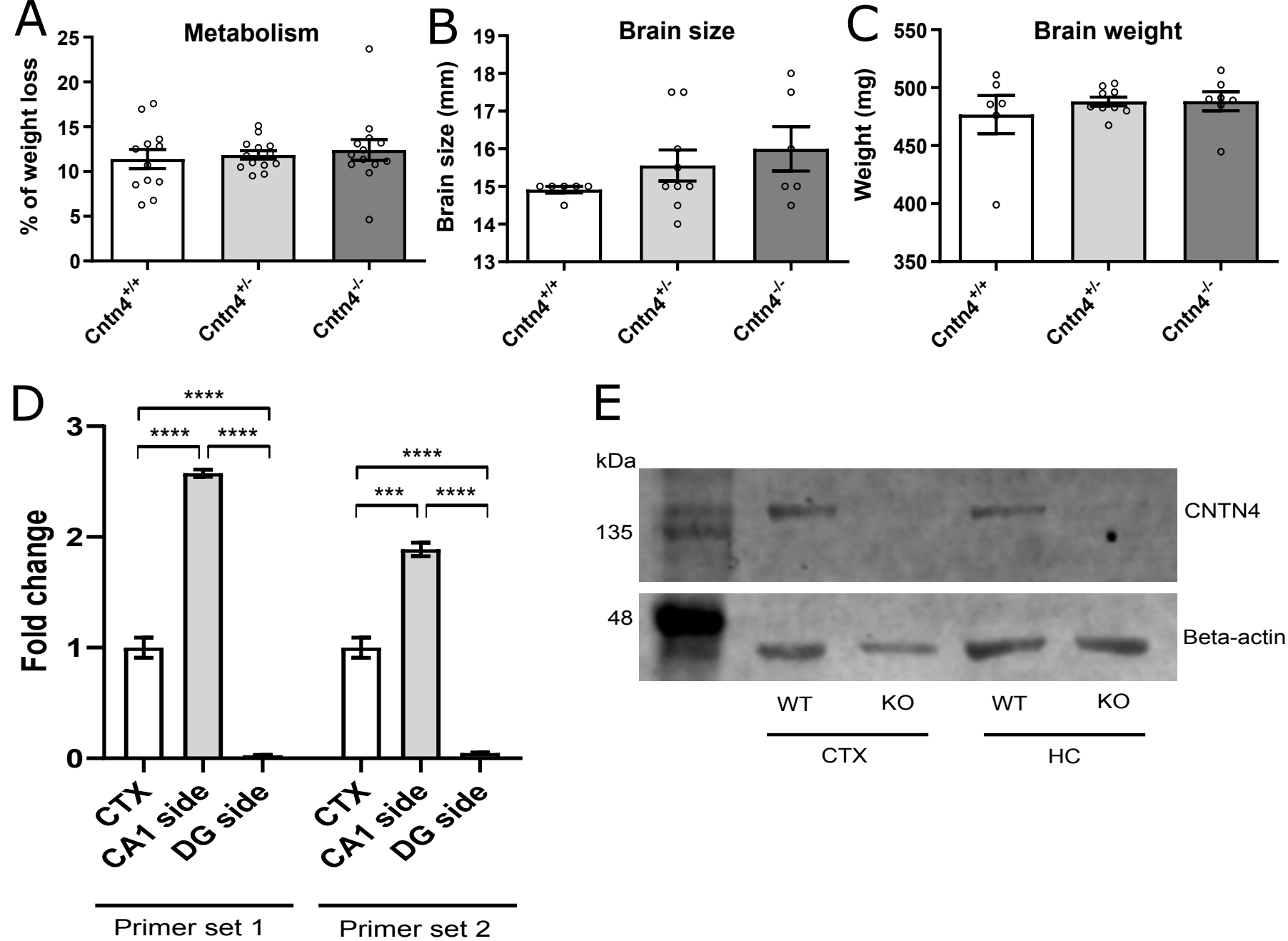

Supplement: Supplementary file 2 — supplementary figure 1 [file 41398_2021_1223_MOESM2_ESM.pdf]

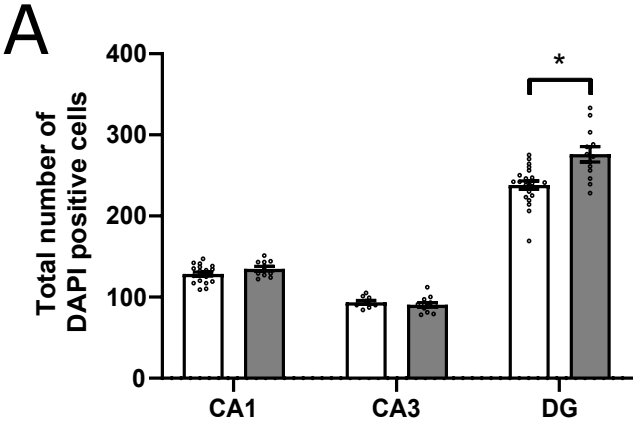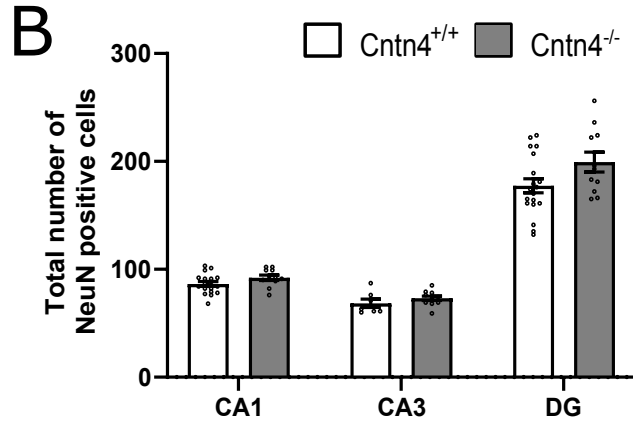

Supplement: Supplementary file 3 — supplementary figure 2 [file 41398_2021_1223_MOESM3_ESM.pdf]

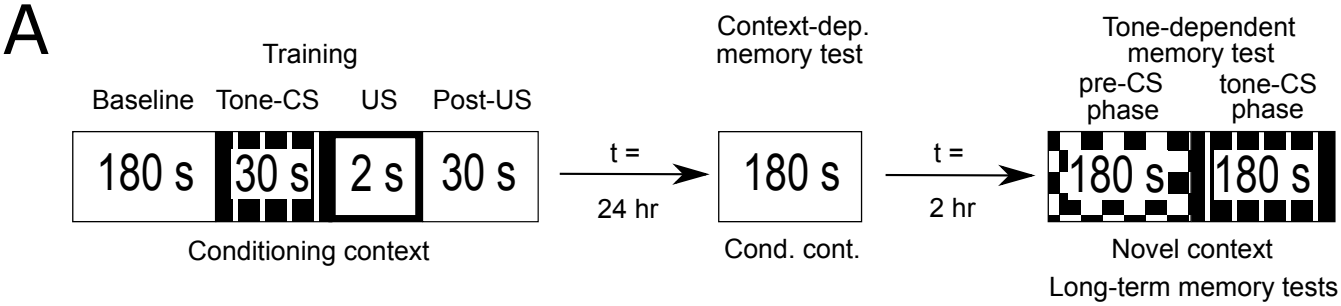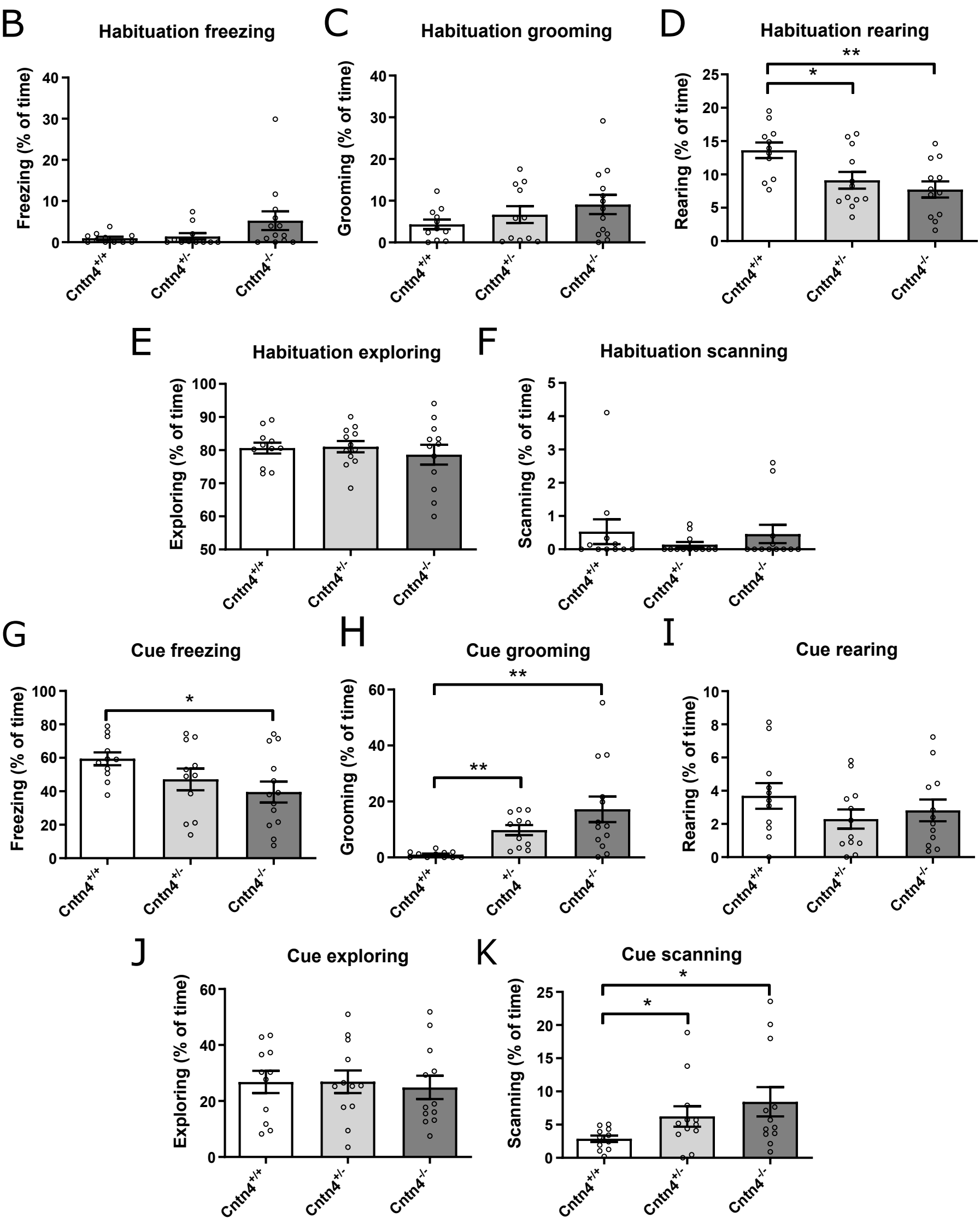

Supplement: Supplementary file 4 — supplementary figure 3 [file 41398_2021_1223_MOESM4_ESM.pdf]
